# Fistule œsotrachéale compliquant un lymphome non hodgkinien B médiastinal primitif: à propos d'un cas

**DOI:** 10.11604/pamj.2019.32.30.17143

**Published:** 2019-01-17

**Authors:** Amine Benmoussa, Mostafa Mechtoune, Rajaa Tissir, Ilias Tazi, Lahoussine Mahmal

**Affiliations:** 1 Service d'Hématologie, CHU Mohammed VI Marrakech, Maroc

**Keywords:** Lymphome médiastinal primitif, fistule trachéo-oesophagienne, pronostic, traitement, Primary mediastinal lymphoma, tracheœsophageal fistula, prognosis, treatment

## Abstract

Le lymphome non hodgkinien médiastinal primitif (LNHMP) est un cancer rare, se complique exceptionnellement par des fistules trachéo-œsophagiennes reliant directement l'œsophage à la trachée, qui sont secondaires à l'atteinte tumorale œsophagienne ou à la chimiothérapie (l'intérêt de notre cas). Nous rapportons le cas d'une patiente de 24 ans, d'origine marocaine, suivie pour LNH médiatisnal primitif à grandes cellules B révélé par une dyspnée avec dysphagie et altération de l'état général. La patiente est mise sous chimiothérapie mais le tableau s'aggrave après la deuxième cure par l'apparition des infections pulmonaires à répétition avec notion de toux lors des repas responsable d'une impossibilité d'alimentation, le diagnostic de fistule trachéo-œsophagienne est retenu par fibroscopie œsogastroduodénale, mais l'évolution est marquée par le décès de la patiente malgré la mise en place de stent endoscopique et la bonne réponse à la chimiothérapie. La découverte précoce d'une fistule trachéo-œsophagienne chez le patient atteint de LNHMP est indispensable permettant la mise en route d'un traitement approprié.

## Abstract (en)

Primary mediastinal non-Hodgkin's lymphoma(PMNHL) is a rare cancer. Exceptionally, it can be complicated by tracheœsophageal fistulas, directly connecting the esophagus and the trachea and secondary to esophageal tumor or chemotherapy (hence the interest of our case). We report the case of a 24-year old Moroccan female patient, treated for primary mediastinal large B cell NHL revealed by dyspnœa associated with dysphagia and alteration of general condition. The patient underwent chemotherapy but her health condition worsened after a second treatment due to the occurrence of recurrent pulmonary infections associated with cough during meals making swallowing impossible. Esogastroduodenal fibroscopy was performed which confirmed the diagnosis of tracheœsophageal fistula. Outcome was marked by patient's death despite endoscopic stent placement and a good response to chemotherapy. Early discovery of tracheœsophageal fistula in patients with PMNHL is essential because it enables the implementation of an appropriate treatment.

## Introduction

Les carcinomes œsophagiens ou pulmonaires primitifs constituent les causes les plus fréquentes des fistules trachéobronchiques secondaires aux tumeurs. Le lymphome non hodgkinien (LNH) est une maladie rare qui touche rarement l'œsophage. Les fistules secondaires aux lymphomes sont rarement décrites dans la littérature. Nous rapportons le cas d'une patiente suivie pour lymphome médiastinal primitif, mise initialement sous chimiothérapie, avec évolution défavorable due au développement de fistule trachéo-œsophagienne.

## Patient et observation

**Figure 1:**
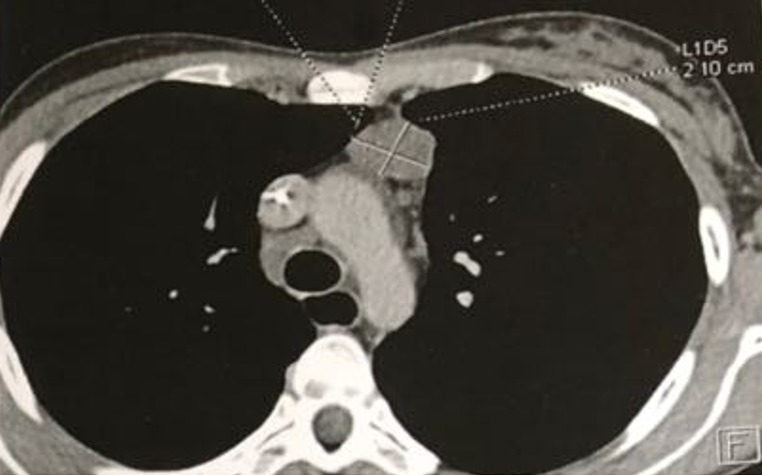
Adénopathie médiatisnale antérieure avec dilatation de l'œsophage à la TDM thoracique

**Figure 2:**
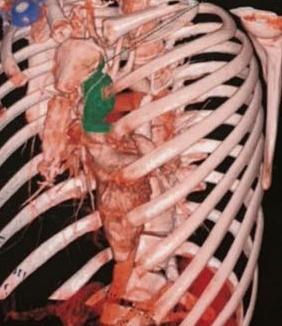
Aspect scannographique de la masse médiatisnale antérieure

Il s'agissait d'une patiente âgée de 24 ans, sans ATCDS pathologiques particuliers, qui s'est présenté pour une symptomatologie d'installation rapidement progressive évoluant depuis 6 semaines, faite de dyspnée au repos, avec dysphagie évoluant dans un contexte d'altération de l'état général (amaigrissement chiffré a 4kg ; asthénie ; anorexie ; fièvre prolongée sans notion des sueurs nocturnes). L'examen clinique trouvait une patiente avec PS (performance staturale): 2, stable sur le plan neurologique, hémodynamique, le reste de l'examen somatique était sans particularités, notamment pas d'adénopathies ni hépatosplénomégalie. La tomodensitométrie (TDM) thoracique a montré la présence d'une masse médiastinale antérieure de 36 x96 x100 mm, non rehaussée par le produit de contraste. La patiente a bénéficié d'une biopsie de la masse médiastinale antérieure qui était en faveur de LNH diffus à grandes cellules B CD20+. Le bilan d'extension (TDM cervico-thoraco-abdomino-pelvienne, Hémogramme, urée, créatinine, Aspartate Aminotransférase, Alanine transaminase) était normal en dehors de l'atteinte médiastinale ainsi que le bilan préchimiothérapie (Echo-cœur et les sérologies VIH, VHC, VHB). La LDH était élevée à 350 UI et la biopsie ostéo-médullaire (BOM) était normale. La patiente a été mise sous protocole RCHOP (Rituximab, Endoxan,Cyclophosphamide, Prednisolone, Doxorubicine). L'évolution était marquée après la 2^ème^ cure de chimiothérapie, par l'apparition des infections pulmonaires à répétition, le tableau s'est aggravé par l'installation d'une dysphagie mixte avec toux lors des repas et cachexie. Une TDM thoracique a été demandée après la 3^ème^ cure, objectivant la présence d'une fistule trachéo-œsophagienne avec des micronodules pulmonaires droits en rapport avec la fistule et régression de la masse médiastinale de >50%. La fibroscopie œsogastroduodénale (FOGD) demandée a confirmé la présence de fistule trachéo-œsophagienne. Une mise en place de stent endoscopique œsophagien a été réalisée, ainsi qu'une sonde de gastrostomie, mais l'évolution était marquée par le décès de la patiente par un choc septique (Figure 1, Figure 2).

## Discussion

Le lymphome B à grandes cellules primitif du médiastin (LBPM) est une entité rare, qui représente moins de 3% detous les lymphomes non hodgkiniens [1], et environ 5% des lymphomes agressifs de l'adulte [2]. C'est un lymphome survenant dans le médiastin antérieur (surtout le thymus), touchant essentiellement les sujets jeunes de sexe féminin, 30 à 40 ans [2]. Ce lymphome se complique rarement de fistule trachéo-œsophagienne. Cette dernière peut être congénitale ou acquise (tumeur, infection, traumatisme local, mais essentiellement après trachéotomie ou ventilation assistée par sonde trachéale). La fistule trachéo-œsophagienne est une complication rare des tumeurs, retrouvée principalement dans le cancer du poumon ou l'œsophage, et marque exceptionnellement l'évolution du lymphome et se présente principalement après une radiothérapie ou chimiothérapie. Sa localisation se situe généralement entre la trachée ou les bronches ou poumon et l'œsophage [3]. Les manifestations cliniques principales de la fistule trachéo-œsophagienne sont la toux paroxystique rythmée par l'alimentation et lors de l'ingestion de liquide, les infections pulmonaires à répétition, l'hémoptysie et les abcès pulmonaires récurrents. La tomodensitométrie thoracique permet de préciser les dégâts parenchymateux et parfois de visualiser la fistule, c'est le cas chez notre patiente. Le diagnostic de la fistule trachéo-œsophagienne est confirmé par la fibroscopie bronchique qui permet de faire le diagnostic et le bilan lésionnel, de déterminer le siège de cette fistule par rapport au larynx et de déterminer l'état des tissus environnants et notamment la présence d'une fibrose de la muqueuse. La fibroscopie bronchique n'a pas été réalisée chez notre patiente. Le diagnostic a été retenu sur un faisceau d'arguments cliniques, scannographies et endoscopiques (FOGD) [4-7]. La fibroscopie œsogastroduodénale (FOGD) peut contribuer au diagnostic et à la prise en charge thérapeutique, comme notre cas présent. L'évolution spontanée sans traitement est fatale avec une survie moyenne d'une à six semaines. L'objectif de traitement est basé sur la guérison de la fistule et du lymphome. Le stent œsophagien endoscopique est l´approche privilégiée suivie d´une chimiothérapie, qui est difficile en présence des infections pulmonaires et de malnutrition. Le stent œsophagien endoscopique est moins invasif et peut entraîner une guérison de la fistule, la résolution de la septicémie en association avec les antibiotiques et l'amélioration de l´état nutritionnel [8, 9]. Dans le cas actuel, le stent œsophagien n'a pas été réussi. Parmi les autres options, La mise en place d'une sonde de gastrostomie, peut contribuer à l'amélioration de l'état nutritionnel, notre patiente a bénéficié d'une sonde de gastrostomie, mais l'évolution était défavorable.

## Conclusion

La fistule trachéo-œsophagienne est une complication rare du lymphome non hodgkinien. Elle doit être suspectée chez les patients présentant des symptômes inexpliqués, tels qu´une toux et une suffocation sévère, le diagnostic précoce et la mise en place d´un traitement adapté permettent d'améliorer le pronostic et augmenter les chances de survie.

## Conflits d'intérêts

Les auteurs ne déclarent aucun conflit d'intérêt.
